# Mitochondrial biogenesis is rate limiting-factor for axonal growth

**DOI:** 10.1186/2193-1801-4-S1-P25

**Published:** 2015-06-12

**Authors:** Merle Mandel, Annika Vaarmann, Akbar Zeb, Przemyslaw Wareski, Joanna Liiv, Malle Kuum, Eva Antsov, Mailis Liiv, Vinay Choubey, Allen Kaasik

**Affiliations:** Department of Pharmacology, Centre of Excellence for Translational Medicine, Institute of Biomedicine and Translational Medicine, University of Tartu, Estonia

**Keywords:** mitochondrial biogenesis, PGC-1α, neuronal development

During early development neurons undergo complex morphological rearrangements. This active axo-dendritic growth must be supported by sufficient cellular energy, thus, cellular energy status could be a key factor in initiating this process. However, the precise mechanisms of how cells adapt their energetic status to physiological demand and upcoming energy needs and how they are coupled to adaptive energy generation, e.g., increased ATP production by mitochondria, remain unclear. Previous studies have shown that peroxisome-proliferator-activated receptor gamma co-activator 1 (PGC-1α) is a master regulator of mitochondrial biogenesis and cellular energy metabolism. Our aim was to examine whether neuronal growth depends on mitochondrial biogenesis and whether activation of cell growth pathways could promote mitochondrial biogenesis to support the energetic need of neuronal development. Over-expression of PGC-1α in cortical neurons increased mitochondrial density in the periphery of axonal tree and was two-fold higher in axonal tips compared to control group. Moreover, induction of mitochondrial biogenesis by PGC-1α facilitated the axonal growth and neuronal development. Activation of PGC-1α upstream kinases such as Ca2+/calmodulin-dependent protein kinase kinase 2 (CaMKK2), transforming growth factor-β-activated kinase (TAK1) and STE-related adaptor (STRAD) increased PGC-1α transcriptional activity and mitochondrial density in axons. Most importantly, they promoted neuronal development through mitochondrial biogenesis. This study shows that mitochondrial biogenesis itself is limiting factor for axonal growth and AMPK-PGC-1α upstream pathways sensing cellular energy status could signal not only the energy deficit but also upcoming energy need to generate new mitochondria.

